# Enhanced LGMD Model with Adaptive Probabilistic Regulation for Compound Interference

**DOI:** 10.3390/biomimetics11070488

**Published:** 2026-07-11

**Authors:** Hao Luan, Changmiao Nie, Weikun Chen, Bin Yang, Hongwei Li, Jintao Zhao

**Affiliations:** School of Automation and Electrical Engineering, Tianjin University of Technology and Education, Tianjin 300222, China; haoluan@tute.edu.cn (H.L.); cmnie123@foxmail.com (C.N.); 2022120001@tute.edu.cn (W.C.); 2021080001@tute.edu.cn (B.Y.); hongwei_li@tute.edu.cn (H.L.)

**Keywords:** LGMD-inspired, collision detection, probabilistic neural network, spatial residual feedback

## Abstract

When subjected to compound interference, such as spatial noise and high-frequency jitter, current LGMD-inspired collision detection models for micro-robots are prone to false alarms and perceptual degradation. To address this challenge, this paper proposes an enhanced visual perception model that incorporates adaptive Gaussian random variables and spatial residual feedback (SRF). These random variables filter out discrete spatial noise, while the SRF suppresses global image shifts induced by jitter. Evaluations on synthetic and real-world video sequences validate the proposed mechanisms. Comparative results demonstrate that the model effectively reduces false responses under compound interference, thereby maintaining robust success rate (SR), discrimination ratio (DR), and membrane potential stability index (MPSI) metrics. To explain this robustness, ablation analyses further verify the synergistic benefits of the SRF and the Gaussian random variables. Furthermore, statistical results on the random variables indicate that, under compound interference, the adaptive probabilistic model outperforms fixed probabilistic configurations. By ensuring robust collision perception against such interference, this work enhances the practical viability of LGMD-inspired visual systems.

## 1. Introduction

Autonomous navigation of unmanned aerial vehicles and micro-robots relies heavily on reliable collision detection and avoidance mechanisms [1,2,3]. Mainstream deep learning-based vision methods require massive computational resources [4], which are prohibitive for micro-devices limited by size and power consumption [5]. The Lobula Giant Movement Detector (LGMD) in the locust visual system exhibits highly efficient and low-power looming perception capabilities [6]. It provides a valuable reference for constructing lightweight bio-inspired visual neural networks [7,8].

In practical operations, complex environments pose severe challenges to the application of bio-inspired visual models [9,10]. Particularly during locomotion, micro-robots are susceptible to compound interference from high-frequency jitter and spatial noise [10]. These two types of interference operate through fundamentally distinct mechanisms: spatial noise introduces random, discrete pixel variations within a single frame, whereas high-frequency jitter causes global spatial displacement across the entire image sequence. Because most existing LGMD models adopt feedforward architectures [11,12], continuous error accumulation occurs once these dynamic disturbances propagate through the pathways and continuous error accumulation occurs, triggering frequent false positive responses. Even with the addition of auxiliary denoising or filtering modules, it remains difficult to arrest error accumulation without filtering out core motion features associated with true collision risks.

To alleviate the false responses caused by these compound interference, this study designs a visual perception model using spatial residual feedback (SRF) and Gaussian probability distributions. This architecture suppresses interference by separating and filtering them layer by layer. To tackle high-frequency jitter, the SRF generates feedback signals acting on the input end, which reduces the induced global image displacement and thereby simplifies complex compound interferences into spatial noise. Meanwhile, utilizing prior information regarding target brightness, a contrast polarity initialization scheme is employed to shield redundant channels, and random variables following a Gaussian distribution are selectively embedded into the active pathways. Because fixed probability parameters are less adaptable to variable scenarios, this paper further proposes an adaptive parameter tuning method based on initial estimates. Initially, to measure the spatial noise intensity and the high-frequency jitter level, the model calculates dynamic parameters. It then adaptively determines the dominant polarity channel and configures the optimal transmission probability for the activated channel. Through the synergy of feedback buffering and probability filtering, the proposed model maintains continuous target motion contours and enhances noise robustness in complex and variable dynamic environments.

The adaptive Gaussian random variables conform to the physiological principles of insect visual pathways. While previous networks used fixed probability to simulate the stochastic neurotransmitter release at synaptic clefts [13], a static probability limits the adaptability of neural circuits. In biological systems, synaptic transmission is state-dependent and governed by short-term synaptic plasticity and activity-dependent gain control [14]. By tuning the transmission probability based on environmental noise intensity, the model replicates this regulation. This enables visual neuropils, such as the lamina and medulla, to lower synaptic efficacy under severe noise, thereby preventing false activation of the looming detection circuit [15].

Furthermore, the Spatial Residual Feedback (SRF) reflects the top-down centrifugal visual pathways projecting from the lobula complex back to peripheral layers [16]. Neurophysiological evidence indicates that these re-entrant feedback circuits modulate the local spatial summation of presynaptic neurons [17]. The SRF simulates how insect visual circuits convey global background motion dynamics back to ON/OFF channels. This feedback suppresses background shifts induced by high-frequency jitter by modulating presynaptic inputs to subtract background motion. Through the integration of synaptic adaptation and spatial feedback regulation, the model maintains consistent visual perception for localized looming threats.

The main contributions of this paper are summarized as follows:

- We propose an enhanced LGMD-inspired visual perception model that resolves the vulnerability of existing architectures under compound interferences. By the structural synergy between SRF and adaptive Gaussian random variables. A spatial residual feedback mechanism is introduced in the early visual pathways to suppress high-frequency jitter, decoupling the compound interference into discrete spatial noise. Based on this decoupled visual signal, adaptive Gaussian random variables are employed to filter the residual noise, facilitating reliable collision detection.
- We develop an environment-aware adaptive parameter tuning method to overcome the challenge of fixed probabilistic configurations in compound interference. Instead of relying on fixed probability that fail under varying noise levels, our approach extracts spatial noise intensity and jitter levels from initial environmental metrics. This allows the model to calculate and scale the optimal synaptic transmission probability through a predefined mapping function, enhancing tracking reliability under compound interference.
- Extensive experiments are conducted across various datasets to verify the effectiveness of the proposed model. The results demonstrate its robustness in preserving collision features while suppressing compound interference.

The remainder of this paper is organized as follows. Section 2 reviews related studies on visual obstacle avoidance and noise reduction. Section 3 details the architecture of the proposed model. Section 4 introduces experimental setup and datasets. Section 5 presents and analyzes experimental results. Section 6 concludes this paper.

## 2. Related Work

Researchers have continuously optimized the network architecture of Lobula Giant Movement Detector (LGMD) models to improve their robustness against visual interference in complex environments. This section reviews existing studies from two perspectives: interference types and corresponding processing mechanisms.

### 2.1. Spatial Noise Suppression Methods of LGMD Visual Networks

Many existing LGMD visual neural networks adopt deterministic filtering methods to suppress spatial noise. Traditional models typically employ fixed lateral inhibition kernels to filter isolated noise pixels [9,18]. Some studies introduce a dedicated denoising layer (D-layer) before synaptic integration, utilizing local neighborhood pixel expectations and empirical hard thresholds to remove weak signals [19]. Another strategy involves the grouped excitation mechanism, which extracts combined signals of adjacent neurons and discards weak responses using predefined boundaries [20]. Additionally, spatiotemporal lateral inhibition mechanisms are adopted to filter translating background edges, and spatial Gaussian filters are applied at the early visual stage to smooth the input luminance signals [21]. Deterministic models rely on fixed mathematical thresholds. When the visual input contains high-intensity random noise, this noise distorts model outputs. Incapable of adapting to these random variations, deterministic thresholds often cause the models to interpret strong noise as looming threats or discard actual motion features, thereby generating false alarms. This challenge drives the shift toward probabilistic models.

Probabilistic models utilize the inherent randomness of biological synaptic transmission to filter visual noise [22]. For instance, recent studies incorporate Bernoulli-distributed random variables into inter-layer synaptic transmission [12]. By replacing absolute thresholds with random transmission, this approach reduces isolated background noise while retaining continuous collision features. Subsequent models substitute binary distributions with Gaussian random variables to provide a smoother tolerance margin against spatial interference [23]. While these mechanisms process spatial noise, their values of random variables are fixed after offline calibration. Since these fixed variables cannot handle changing environmental noise densities, there is a need for probabilistic models that can dynamically adjust their random variables to adapt to specific environmental states. Moreover, processing spatial noise only resolves discrete pixel interference. When deployed on micro-robots, locomotion-induced high-frequency jitter causes global pixel displacements across consecutive frames. Lacking internal feedback mechanisms to suppress these dynamic shifts, feedforward probabilistic models continuously amplify such jitter-driven errors.

Despite the progress in suppressing spatial noise through probabilistic frameworks, existing methods are susceptible to compound interference, where discrete spatial noise and jitter occur simultaneously. The random variables in current probabilistic models are typically fixed after calibration, preventing adaptability to changing environmental noise densities. Moreover, suppressing spatial noise only addresses discrete pixel interference. High-frequency jitter during locomotion causes global pixel displacements across consecutive frames. Since feedforward models lack internal feedback mechanisms to compensate for such shifts, jitter-induced errors tend to accumulate.

To address these interferences, the proposed model introduces a neural structure to process complex visual signals. The adaptive Gaussian random variables filter discrete spatial noise by scaling synaptic transmission probabilities according to ambient noise intensity. Concurrently, the Spatial Residual Feedback (SRF) handles high-frequency jitter by conveying background motion back to the ON/OFF channels, which subtracts background shifts. This approach mitigates the impact of compound interference while maintaining robust looming perception.

### 2.2. High-Frequency Jitter Suppression Methods of LGMD Visual Networks

To address high-frequency jitter during micro-robot navigation, conventional approaches typically rely on external stabilization. Physical solutions, such as gimbals, isolate the camera from carrier vibrations but introduce additional payload and energy consumption [24,25], restricting their deployment on lightweight micro-robots. Electronic video stabilization algorithms use feature point matching or optical flow tracking to compensate for global image shifts [26,27]. These pixel-level matching procedures consume substantial computational resources [28,29]. Consequently, this inherent complexity conflicts with the low-power and high-speed requirements of bio-inspired vision systems [3].

Within the domain of LGMD networks, internal jitter suppression mechanisms remain sparse. Most classic models operate under the assumption of a stable camera, generating frequent false alarms when subjected to global pixel displacements [30,31]. Recently, a dynamic temporal variance (DTV) feedback mechanism is introduced to regulate the visual network under jitter [32]. By calculating the temporal variance of pixel intensities from historical frames, this model dynamically adjusts its inhibition thresholds. While this feedback pathway effectively suppresses high-frequency jitter, it is sensitive to spatial noise. In real-world scenarios, visual systems frequently encounter compound interference where high-frequency jitter is coupled with dense spatial noise. In such compound environments, the model’s anti-jitter capability is significantly weakened as random spatial noise distorts the underlying temporal variance calculations.

In summary, most existing methods struggle to simultaneously achieve spatial noise suppression and jitter buffering within a unified computing structure. To further improve perceptual robustness, it is fundamental to break the constraints of feedforward architectures and equip probabilistic models with dynamic environmental adaptability. Accordingly, a network coupling adaptive transmission probabilities and spatial residual feedback is proposed in Section 3 to address the challenge of compound interference.

## 3. Formulation of the Proposed Model

**Figure 1:**
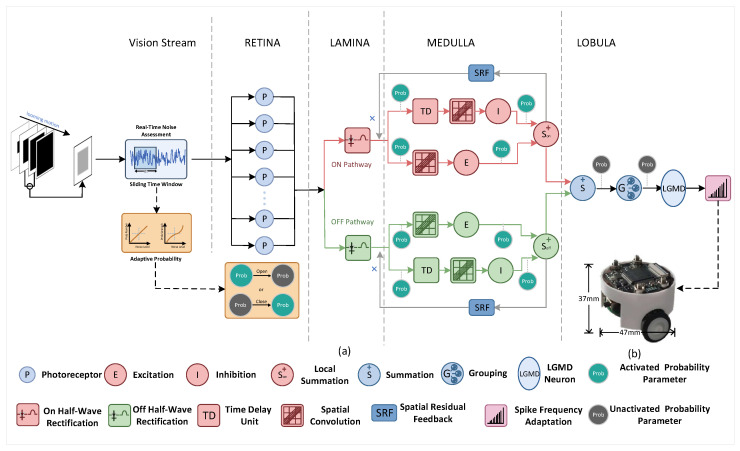
Architecture of the proposed enhanced LGMD visual network featuring spatial noise filter and high-frequency jitter suppression mechanisms. Panel (**a**) illustrates the overall feedforward signal processing pathways of the model. In the diagram, solid black arrows represent the primary visual signal flow, while dashed black arrows indicate the adaptive probability modulation flow and the final control output. Solid red and green arrows denote the distinct signal processing streams within the ON and OFF pathways, respectively. Solid grey arrows trace the Spatial Residual Feedback (SRF) loops, and dashed grey lines indicate the application of probability parameters (Prob) to specific processing nodes. Panel (**b**) displays the custom-developed mobile micro-robot platform utilized to record the video sequences for experimental evaluation, for future deployment of the proposed model.

The proposed LGMD visual network features adaptive random variables and SRF that work in concert. As depicted in the overall architecture (Figure 1), adaptive random variables suppress spatial noise, while SRF targets high-frequency jitter. A contrast polarity initialization method and an interference evaluation method at the network front end provide the foundation for adjusting synaptic transmission probabilities. This approach replaces fixed probability parameters transmission paradigms and enables the model to handle compound interferences.

The model incorporates Gaussian random variables into the ON/OFF dual-channel network. Transmission probabilities adjust in response to interference hyperparameters derived from the input video. Adaptive random variables provide a dynamic modulation approach, ensuring the model maintains a stable response intensity across varying compound interference. The SRF mechanism targets high-frequency jitter. It operates through feedback loops within the ON and OFF channels. By performing multi-scale operations, SRF calculates feature residuals and generates a regulatory mask. This mask buffers sudden spatial offsets induced by global frame displacement.

### 3.1. Computational Retina Layer

The retina layer mimics photoreceptor sensitivity to transient luminance changes. For a discrete video sequence, the photoreceptor output P(i,j,t) at coordinate (i,j) in the *t*-th time step represents the grayscale difference between adjacent frames.

(1) P(i,j,t)=L(i,j,t)−L(i,j,t−1)

where L(i,j,t) denotes the normalized luminance value of the corresponding pixel in the current frame.

### 3.2. Computational Lamina Layer

The lamina layer receives differential signals from the retina and separates them into parallel ON and OFF channels. This layer applies negative feedback regulatory masks, $M_{\mathrm{on}}$ and $M_{\mathrm{off}}$, from the spatial residual feedback loop to suppress high-frequency jitter.

(2) $$P_{\mathrm{on}}\left(i,j,t\right)={\left[P\left(i,j,t\right)⊙M_{\mathrm{on}}\left(i,j,t\right)\right]}^{+}$$

(3) $$P_{\mathrm{off}}\left(i,j,t\right)={\left[P\left(i,j,t\right)⊙M_{\mathrm{off}}\left(i,j,t\right)\right]}^{-}$$

where ${\left[\cdot \right]}^{+}$ and ${\left[\cdot \right]}^{-}$ denote positive and negative half-wave rectifications, respectively. The operator ⊙ denotes element-wise multiplication, and $M_{\mathrm{on}}$ and $M_{\mathrm{off}}$ represent the feedback regulatory masks for the two corresponding channels.

### 3.3. Computational Medulla Layer

The polarity-separated signals enter the medulla layer for spatiotemporal feature extraction. Parallel excitatory and inhibitory layers simulate the attenuation and random properties of neural signals.

#### 3.3.1. Excitation Layer

The network extracts center-excitatory features of the local receptive field using 2D Gaussian spatial convolution. Gaussian random variables modulate the signal transmission.

(4) $$E_{\mathrm{on}}\left(i,j,t\right)=\sum_{p=-R_{e}}^{R_{e}}\sum_{q=-R_{e}}^{R_{e}}P_{\mathrm{on}}\left(i+p,j+q,t\right)X_{E_{\mathrm{on}}}\left(i,j\right)G_{\sigma 1}\left(p,q\right)$$

(5) $$E_{\mathrm{off}}\left(i,j,t\right)=\sum_{p=-R_{e}}^{R_{e}}\sum_{q=-R_{e}}^{R_{e}}P_{\mathrm{off}}\left(i+p,j+q,t\right)X_{E_{\mathrm{off}}}\left(i,j\right)G_{\sigma 1}\left(p,q\right)$$

where $P_{\mathrm{on}}$ and $P_{\mathrm{off}}$ denote the polarity signals. $G_{\sigma 1}$ represents the Gaussian spatial convolution kernel. *p* and *q* are the coordinate offsets bounded by the receptive field radius $R_{e}$. The variables $X_{E_{\mathrm{on}}}$ and $X_{E_{\mathrm{off}}}$ represent synaptic transmission probabilities. They are independent and identically distributed across all pixels and frames. The system samples these variables from a truncated Gaussian distribution $N(\mu ,\sigma^{2})$ bounded within [0,1].

#### 3.3.2. Inhibition Layer

The inhibitory pathway applies a temporal delay while preserving the spatial structure. An exponentially decaying function ψ(l) models this temporal delay.

(6) $$I_{\mathrm{on}}\left(i,j,t\right)=\sum_{p=-R_{i}}^{R_{i}}\sum_{q=-R_{i}}^{R_{i}}\sum_{l=0}^{N_{d}}P_{\mathrm{on}}\left(i+p,j+q,t-1-l\right)X_{I_{\mathrm{on}}}\left(i,j\right)G_{\sigma 2}\left(p,q\right)\psi \left(l\right)$$

(7) $$I_{\mathrm{off}}\left(i,j,t\right)=\sum_{p=-R_{i}}^{R_{i}}\sum_{q=-R_{i}}^{R_{i}}\sum_{l=0}^{N_{d}}P_{\mathrm{off}}\left(i+p,j+q,t-1-l\right)X_{I_{\mathrm{off}}}\left(i,j\right)G_{\sigma 2}\left(p,q\right)\psi \left(l\right)$$

(8) $$\psi \left(l\right)=2e^{-\pi l^{2}}$$

where $R_{i}$ specifies the inhibitory receptive field radius. $N_{d}$ defines the maximum temporal delay frames. *l* represents the current frame offset. $G_{\sigma 2}$ denotes the Gaussian spatial convolution kernel for the inhibitory pathway. The variables $X_{I_{\mathrm{on}}}$ and $X_{I_{\mathrm{off}}}$ represent the synaptic transmission probabilities for this layer.

#### 3.3.3. Local Signal Integration

The medulla terminal integrates the excitatory and delayed inhibitory signals for each channel.

(9) $$S_{\mathrm{on}}\left(i,j,t\right)={\left[E_{\mathrm{on}}\left(i,j,t\right)X_{SE_{\mathrm{on}}}\left(i,j\right)-\alpha_{1}I_{\mathrm{on}}\left(i,j,t\right)X_{SI_{\mathrm{on}}}\left(i,j\right)\right]}^{+}$$

(10) $$S_{\mathrm{off}}\left(i,j,t\right)={\left[E_{\mathrm{off}}\left(i,j,t\right)X_{SE_{\mathrm{off}}}\left(i,j\right)-\alpha_{2}I_{\mathrm{off}}\left(i,j,t\right)X_{SI_{\mathrm{off}}}\left(i,j\right)\right]}^{+}$$

where $\alpha_{1}$ and $\alpha_{2}$ denote the inhibitory control coefficients. The variables $X_{SE_{\mathrm{on}/\mathrm{off}}}$ and $X_{SI_{\mathrm{on}/\mathrm{off}}}$ represent the random variables for these pathways. They remain fixed at 1 in the standard configuration and are modulated only during structural ablation studies.

### 3.4. Computational Lobula Layer

#### 3.4.1. Spatial Residual Feedback Pathway

The SRF pathway suppresses high-frequency jitter. It extracts temporal variance from the active polarity channel to construct a feedback mask M(i,j,t). This mask modulates local neural responses in the lamina layer.

The mechanism computes the absolute difference between adjacent frames within a short sliding time window of length $N_{win}=4$. To optimize the trade-off between jitter suppression and detection latency, a monotonically increasing weight vector w=[0.1,0.2,0.3,0.4] scales these temporal differences, assigning greater significance to recent frames. This weighting strategy effectively buffers high-frequency jitter without introducing the response lag typical of uniform averaging methods. The resulting variable, $D_{\mathrm{raw}}\left(i,j,t\right)$, quantifies local signal variations at each spatial coordinate (i,j) and time step *t*.

(11) $$D_{\mathrm{raw}}\left(i,j,t\right)=\sum_{\tau =1}^{N_{win}}w_{\tau }\left|S_{\mathrm{on}/\mathrm{off}}\left(i,j,t-\tau +1\right)-S_{\mathrm{on}/\mathrm{off}}\left(i,j,t-\tau \right)\right|$$

where $S_{\mathrm{on}/\mathrm{off}}$ represents the accumulated signal of the active polarity channel. The index τ denotes the temporal offset within the sliding window.

A logarithmic mapping amplifies weak variations in $D_{\mathrm{raw}}\left(i,j,t\right)$ to generate $D_{\log}\left(i,j,t\right)$. A spatial mean filter then extracts the neighborhood trend to form a baseline $D_{\mathrm{base}}\left(i,j,t\right)$.

(12) $$D_{\log}\left(i,j,t\right)=\log\left(1+10\cdot D_{\mathrm{raw}}\left(i,j,t\right)\right)$$

(13) $$D_{\mathrm{base}}\left(i,j,t\right)=\Phi_{\mathrm{avg}}\left(D_{\log}\left(i,j,t\right)\right)$$

where $\Phi_{\mathrm{avg}}$ denotes a 3×3 spatial average pooling with a stride of 1. This local averaging suppresses high-frequency spatial details. Consequently, $D_{\mathrm{base}}\left(i,j,t\right)$ retains only low-frequency motion characteristics. Subtracting this baseline from the logarithmic signal isolates the high-frequency residual $D_{\mathrm{jitter}}\left(i,j,t\right)$.

(14) $$D_{\mathrm{jitter}}\left(i,j,t\right)=\max\left(0,D_{\log}\left(i,j,t\right)-D_{\mathrm{base}}\left(i,j,t\right)\right)$$

Large looming targets exhibit strong spatial coherence and fall within the baseline $D_{\mathrm{base}}\left(i,j,t\right)$. The residual $D_{\mathrm{jitter}}\left(i,j,t\right)$ isolates the high-frequency variations.

To clarify the functional boundaries and stability of this extraction mechanism, the minimum and maximum limits of the jitter residual are mathematically defined as:

(15) $$D_{jitter_{min}}\left(i,j,t\right)=0,\;\mathrm{if}\;D_{log}\left(i,j,t\right)\leq D_{base}\left(i,j,t\right)$$

(16) $$D_{jitter_{max}}\left(i,j,t\right)=\sup\left(D_{log}\left(i,j,t\right)\right)-\inf\left(D_{base}\left(i,j,t\right)\right)$$

The boundary condition $D_{jitter_{min}}\left(i,j,t\right)=0$ proves that large looming targets with strong spatial coherence do not generate residual errors, ensuring that genuine collision cues are fully preserved. Conversely, $D_{jitter_{max}}\left(i,j,t\right)$ captures the upper bound of high-frequency variations exclusively induced by high-frequency jitter. By restricting the residual within this defined interval $\left[0,D_{jitter_{max}}\right]$, the model isolates dynamic instability without risking computational divergence in subsequent layers.

Subsequently, to scale the extracted residual into the [0,1] interval for the probabilistic regulation, min-max normalization is applied. Using the established boundary values, the normalized spatial residual $D_{norm}\left(i,j,t\right)$ is formulated as

(17) $$D_{norm}\left(i,j,t\right)=\frac{D_{jitter}\left(i,j,t\right)-D_{jitter_{min}}\left(i,j,t\right)}{D_{jitter_{max}}\left(i,j,t\right)-D_{jitter_{min}}\left(i,j,t\right)}$$

A Sigmoid function maps the normalized residual into the feedback mask M(i,j,t).

(18) $$M\left(i,j,t\right)=\frac{1}{1+\exp(-\left(D_{\mathrm{norm}}\left(i,j,t\right)-0.5\right))}$$

where $D_{\mathrm{norm}}\left(i,j,t\right)$ is the min-max normalized representation of $D_{\mathrm{jitter}}\left(i,j,t\right)$ bounded in [0,1]. This mask M(i,j,t) provides negative feedback to mitigate jitter-induced displacements in the early visual processing stage.

#### 3.4.2. Summation Layer

The summation layer integrates the ON and OFF polarity signals.

(19) $$S\left(i,j,t\right)=S_{\mathrm{on}}\left(i,j,t\right)+S_{\mathrm{off}}\left(i,j,t\right)$$

This merged signal serves as the input for the subsequent grouping layer.

#### 3.4.3. Grouping Layer

The grouping layer extracts continuous expanding edges of looming targets and suppresses isolated noise. It calculates a transmission coefficient $C_{e}$ using a 3×3 weight matrix $W_{g}$. The maximum coefficient in the current frame determines the global signal gain ω(t). The layer modulates the summation signal using $C_{e}$ and ω(t) to generate the grouped feature G(i,j,t). A threshold $T_{s}$ then truncates this feature to enforce spatial coherence.

(20) $$C_{e}\left(i,j,t\right)=\sum_{p=-1}^{1}\sum_{q=-1}^{1}S\left(i+p,j+q,t\right)X_{G}\left(i,j\right)W_{g}\left(p,q\right)$$

(21) $$\omega \left(t\right)=\max_{\left(x,y\right)}\left\{C_{e}\left(x,y,t\right)\right\}C_{w}^{-1}+0.01$$

(22) $$G\left(i,j,t\right)=S\left(i,j,t\right)C_{e}\left(i,j,t\right)\omega {\left(t\right)}^{-1}$$

(23) $$G\left(i,j,t\right)=\left\{\begin{array}{cc} G(i,j,t), & \mathrm{if}\;G\left(i,j,t\right)\geq T_{s}\\ 0, & \mathrm{otherwise} \end{array}\right.$$

where $W_{g}$ represents the local weight matrix, $C_{w}$ is the global gain adjustment constant, and $T_{s}$ denotes the spatial filtering threshold. The parameter $X_{G}$ represents the random variable for this layer. It remains fixed at 1 in the standard configuration and is modulated only during structural ablation studies.

#### 3.4.4. The LGMD Cell

The network spatially integrates the grouped features to compute the global total excitation $K_{\mathrm{sum}}\left(t\right)$ at time step *t*. A sigmoid function maps this excitation to the normalized membrane potential K(t), simulating the nonlinear saturation of biological neurons.

(24) $$K_{\mathrm{sum}}\left(t\right)=\sum_{i=1}^{R}\sum_{j=1}^{C}G\left(i,j,t\right)$$

(25) $$K\left(t\right)=\frac{1}{1+\exp\left(-\frac{K_{\mathrm{sum}}\left(t\right)}{R\cdot C}\right)}$$

where *R* and *C* represent the row and column dimensions of the input video frame, respectively.

#### 3.4.5. Spike Frequency Adaptation

To amplify the neural responses of true collision features, we introduce a spike frequency adaptation (SFA) module based on nonlinear momentum integration [33,34]. This module captures continuous looming stimuli and nonlinearly amplifies the sustained excitation signals.

(26) $$V_{\mathrm{base}}\left(t\right)=V_{\mathrm{base}}\left(t-1\right)+\delta \left[K\left(t\right)-V_{\mathrm{base}}\left(t-1\right)\right]$$

(27) $$\varepsilon \left(t\right)=\max\left(0,K\left(t\right)-V_{\mathrm{base}}\left(t\right)\right)$$

(28) F(t)=βF(t−1)+ε(t)

(29) $$K_{\mathrm{sfa}}\left(t\right)=K\left(t\right)+\left(1-K\left(t\right)\right)\tanh\left(g\cdot F{\left(t\right)}^{3}\right)$$

where K(t) denotes the initial membrane potential at the *t*-th frame, and $K_{\mathrm{sfa}}\left(t\right)$ represents the amplified output, and *g* is the amplification gain parameter.

The module selectively amplifies targets that provide sustained visual stimulation. First, $V_{\mathrm{base}}\left(t\right)$ tracks the dynamic baseline to establish a suppression threshold. Only active signals exceeding this baseline generate a positive residual ε(t).

The momentum state F(t) acts as a temporal accumulator for this residual. For true looming targets, the continuous expansion ensures a steady influx of ε(t), allowing the momentum to build up rapidly despite the decay coefficient β. The cubic term $F{\left(t\right)}^{3}$ disproportionately amplifies this accumulated excitation. The dynamic margin (1−K(t)) and the tanh function ensure this amplified potential $K_{\mathrm{sfa}}\left(t\right)$ smoothly saturates below 1.

## 4. Experiments Setting

### 4.1. Parameters of the System

The baseline parameter settings of the proposed neural model are listed in Table 1. To ensure a fair and rigorous comparison with existing LGMD-inspired models, foundational parameters are intentionally adopted from established empirical configurations. By keeping these legacy variables constant, we effectively isolate the experimental variables.

To accommodate the varying dynamic ranges of raw optical flow in complex compound interference scenarios, several baseline parameters (e.g., the global gain constant $C_{w}$) are globally pre-calibrated and constrained within a fixed stable range.

### 4.2. Datasets

To evaluate the proposed model, we constructed a visual testing dataset encompassing controlled environments and unconstrained real-world scenes. All sequences are standardized to a spatial resolution of 320×240 pixels at 30 fps. The dataset comprises three primary subsets and a held-out validation set. The held-out validation set covers the three compound interference scenarios detailed in Table 2 and is used exclusively to validate the adaptive modulation strategy in subsequent experiments. Subset 1: This subset contains indoor sequences recorded via a tripod-mounted camera, featuring black and white micro-robots approaching at three distinct speeds. To simulate complex environmental degradation, we synthetically inject multiple interference levels into these clips: Gaussian noise (GNV ∈[0.01,0.12]), salt-and-pepper noise (PNR ∈[0.01,0.12]), and high-frequency jitter (with displacements of 4 to 10 pixels). This subset benchmarks the baseline performance and noise robustness of the model.

Subset 2: These sequences are captured by a custom-developed mobile micro-robot (47 mm × 47 mm × 37 mm) platform [35]. As depicted in Figure 1b, the robot is equipped with an OV5640 CMOS camera module that outputs raw, unstabilized video streams. The robot navigates a desktop at speeds of 3, 5, and 7 cm/s, approaching two static obstacles: a white cup against a black background and a black cup against a white background. Background marker analysis reveals that the mechanical jitter magnitude scales with the moving speed, measuring 2–3 pixels at 3 cm/s, 4–6 pixels at 5 cm/s, and 8–10 pixels at 7 cm/s.

Subset 3: This subset features outdoor sequences recorded using an Insta360 X4 camera (Insta360, Shenzhen, China). All digital stabilization algorithms are intentionally disabled to preserve genuine motion dynamics. It includes three distinct collision scenarios: one stationary camera sequence capturing an approaching human subject and two dynamic handheld sequences navigating toward a stationary black car and a basketball hoop. To rigorously test the model in unstructured environments, we superimpose six progressive levels of Gaussian noise (GNV ∈[0.01,0.06]) and salt-and-pepper noise (PNR ∈[0.01,0.06]).

A full overview of environments, experimental setups and total sample quantities across the three subsets is summarized in Table 2.

To maintain the temporal integrity of the looming stimuli, our dataset protocol is defined at the video-sequence level. Positive samples are categorized as the later video segments exhibiting clear target expansion prior to the actual collision. We utilize the early frames of these looming sequences, where the approaching object has not yet shown obvious expansion and the distance remains relatively stable, as implicit negative conditions. This approach effectively evaluates the model’s resistance to false alarms during non-looming phases. The overall sequence lengths vary naturally depending on the physical looming duration of each recording.

Regarding the computational efficiency of the models is benchmarked offline on a PC platform (AMD Ryzen 5 4600H CPU @ 3.00 GHz (AMD, Santa Clara, CA, USA), 16.0 GB RAM). To evaluate real-time performance, processing latency and frames per second (FPS) are calculated on 40 test sequences sampled from Subset 1 (detailed in Table 2). We selected 4 distinct scene groups, with each group tested under 10 varying conditions: independent Gaussian noise (GNV ∈[0.01,0.1]), salt-and-pepper noise (PNR ∈[0.01,0.12]), high-frequency jitter (with displacements of 1 to 10 pixels), compound spatial noise, and full compound interference (combined spatial noise and jitter). Across these varied noisy conditions, the evaluation results indicate that the proposed model achieves an average processing time of 10.75 ms/frame, corresponding to an operating speed of 93 FPS. Although the introduction of the adaptive mechanisms adds a minor computational overhead compared to other models, this processing speed exceeds standard visual sampling requirements, thereby validating its real-time feasibility for micro-robotic deployment.

### 4.3. Noise Simulation

This section describes the synthesis of spatial noise and high-frequency jitter. These simulated conditions replicate physical visual degradation. They provide a consistent basis for evaluating model stability under compound interference.

#### 4.3.1. Spatial Noise Injection

Realistic spatial noise combines Gaussian and salt-and-pepper noise. In physical environments, these two interferences often coexist due to thermal sensor variations and transmission pixel dropouts. The noise injection proceeds sequentially. The model first applies Gaussian noise to the original frame to simulate continuous electronic interference. It then applies salt-and-pepper noise to simulate discrete defective pixels.

(30) $$F_{\mathrm{gauss}}\left(x,y\right)=\mathrm{clip}\left(F\left(x,y\right)+N\left(0,\sigma_{\mathrm{noise}}^{2}\right),0,1\right)$$

where F(x,y) represents the normalized original pixel value.

(31) $$F_{\mathrm{noisy}}\left(x,y\right)=\left\{\begin{array}{cc} 1, & \mathrm{with}\;\mathrm{probability}\;p_{s}\\ 0, & \mathrm{with}\;\mathrm{probability}\;p_{p}\\ F_{\mathrm{gauss}}\left(x,y\right), & \mathrm{with}\;\mathrm{probability}\;1-p_{s}-p_{p} \end{array}\right.$$

#### 4.3.2. High-Frequency Jitter Injection

A synthetic dataset simulates high-frequency mechanical vibrations. Rather than applying random shifts, this method accounts for the mass damping effect of the camera carrier. This yields realistic motion trajectories.

The maximum displacement amplitude $A_{\mathrm{limit}}$ depends on a baseline displacement $D_{\max}$ and an intensity coefficient γ:

(32) $$A_{\mathrm{limit}}=\gamma \cdot D_{\max}$$

Adjusting the intensity coefficient γ quantitatively generates video sequences with varying disturbance levels.

Under this amplitude constraint, the final jitter displacement trajectory Tr(t) superimposes a low-frequency drift component d(t) and a high-frequency random component. Physical inertia modulates this random component:

(33) $$Tr\left(t\right)=d\left(t\right)+G(n\left(t\right),\sigma_{d})$$

where d(t) simulates the natural low-frequency drift caused by camera motion. n(t) denotes high-frequency random noise. These two components fall within the proportional range permitted by $A_{\mathrm{limit}}$. The operator G denotes Gaussian smoothing with kernel parameter $\sigma_{d}$. This parameter determines the smoothness of trajectory transitions. A smaller $\sigma_{d}$ corresponds to weaker damping and more aggressive responses to high-frequency vibrations.

These experiments simulate micro-robot jitter by setting the inertial parameter $\sigma_{d}\approx 1.2$. Affine transformations map these generated trajectories onto the original frames, yielding the final high-frequency jitter dataset.

### 4.4. Performance Evaluation

This section defines the quantitative metrics used to evaluate model robustness against spatial noise and high-frequency jitter.

#### 4.4.1. Evaluation Metrics

In this study, success rate (SR), discrimination ratio (DR), and membrane potential stability index (MPSI) are selected as the primary evaluation metrics because they directly quantify the model’s signal-level robustness and physical resistance against the targeted compound interferences.

Success rate (SR) measures the overall collision detection reliability across all tested looming stimuli:

(34) $$\mathrm{SR}=\frac{N_{\mathrm{success}}}{N_{\mathrm{total}}}\times 100\%$$

where $N_{\mathrm{success}}$ represents the number of trials where the membrane potential exceeds the predefined warning threshold prior to the actual collision. $N_{\mathrm{total}}$ denotes the total number of tested looming sequences.

The discrimination ratio (DR) evaluates the capacity to distinguish genuine collision signals from non-collision noise. It reflects robustness against spatial interference:

(35) $$\mathrm{DR}=\kappa_{\max}-{\bar{\kappa }}_{\mathrm{noise}}$$

where $\kappa_{\max}$ denotes the peak membrane potential at the collision frame ($i_{\max}$). ${\bar{\kappa }}_{\mathrm{noise}}$ is the average membrane potential during the non-collision phase, calculated as $\frac{1}{T-1}\sum_{i\neq i_{\max}}\kappa \left(i\right)$, where κ(i) is the instantaneous potential at frame *i*, and *T* is the total number of frames. A higher DR indicates a distinct collision peak against background variations.

The membrane potential stability index (MPSI) quantifies response smoothness and peak sensitivity. It reflects resistance to jitter interference:

(36) $$\mathrm{MPSI}=\frac{\kappa_{\max}}{{\bar{\kappa }}_{\mathrm{seq}}}$$

where ${\bar{\kappa }}_{\mathrm{seq}}$ is the mean membrane potential across the entire sequence, defined as $\frac{1}{T}\sum_{i=1}^{T}\kappa \left(i\right)$. A higher MPSI signifies greater overall robustness and a stable potential profile against jitter.

#### 4.4.2. Assessment of Spatial Noise

In natural visual scenes, global variance captures both environmental noise and intrinsic structural variations. Complex edges and textures often dominate this variance. Therefore, evaluating weak discrete noise requires a targeted approach. A high-order Laplacian high-pass filter decouples scene structures from spatial interference.

A 3×3 Laplacian mask applies to the input frame F(x,y) via 2D convolution:

(37) $$K_{\mathrm{lap}}=\left[\begin{array}{ccc} 1 & -2 & 1\\ -2 & 4 & -2\\ 1 & -2 & 1 \end{array}\right]$$

This mask yields a near-zero response to smooth backgrounds and low-frequency edges. It produces strong impulse responses primarily at spatially isolated, high-frequency abrupt changes, such as discrete noise pixels.

By leveraging this frequency response, the model estimates the absolute noise intensity $\hat{\sigma }$ using the global mean of the convolved image:

(38) $$\hat{\sigma }=\frac{\sqrt{\frac{\pi }{2}}}{6(W-2)(H-2)}\sum_{x}\sum_{y}\left|F\left(x,y\right)*K_{\mathrm{lap}}\right|$$

where *W* and *H* represent the frame dimensions. The operator ∗ denotes 2D convolution. The constant coefficient $\sqrt{\pi /2}/6$ derives from Immerkaer’s unbiased estimation theory for Gaussian noise. $\hat{\sigma }$ undergoes mathematical mapping and normalization to integrate into the adaptive probability mechanism.

The final spatial noise hyperparameter $H_{\mathrm{space}}$ is bounded within [0.1,0.9] to maintain mechanism stability under extreme disturbances:

(39) $$H_{\mathrm{space}}=0.1+0.8\times \max\left(0,\min\left(1,\frac{\hat{\sigma }}{{\hat{\sigma }}_{\max}}\right)\right)$$

where the empirical truncation threshold ${\hat{\sigma }}_{\max}$ is set to 100. Under this quantification, $H_{\mathrm{space}}$ correlates directly with the density of spatial noise pixels. A larger $H_{\mathrm{space}}$ (approaching 0.9) indicates high noise density, whereas a value near 0.1 implies a clean visual scene.

#### 4.4.3. Assessment of High-Frequency Jitter

Evaluating robustness against high-frequency jitter requires quantifying the dynamic disturbance intensity. The optical flow extracts the global background trajectory Tr(t). Successive discrete differences of position, velocity V(t), and acceleration A(t) yield the frame-level jerk J(t). This isolates high-frequency jitter from smooth background shifts:

(40) $$\begin{array}{cc} V(t) & =Tr(t)-Tr(t-1)\\ A(t) & =V(t)-V(t-1)\\ J(t) & =A(t)-A(t-1) \end{array}$$

where V(t) represents inter-frame velocity and A(t) denotes acceleration. By computing this third-order difference, J(t) filters out natural low-frequency global translations and captures pure high-frequency jitter.

The local jitter score J(t) utilizes the root mean square of the jerk magnitude within a sliding window of size $N_{\mathrm{win}}$:

(41) $$J\left(t\right)=\sqrt{\frac{1}{N_{\mathrm{win}}}\sum_{\tau =1}^{N_{\mathrm{win}}}{∥J\left(t+\tau \right)∥}^{2}}$$

Normalization maps this metric into the jitter hyperparameter $H_{\mathrm{jitter}}$. This provides a quantitative environmental input for the probabilistic transmission mechanism.

#### 4.4.4. Contrast Polarity Initialization

The system executes contrast polarity initialization during early processing. This limits computational overhead and suppresses non-target background noise. Instead of continuously computing both ON and OFF pathways, the system selectively retains a single unipolar channel matching the looming target’s contrast.

This initialization relies on the temporal luminance variation within the central visual field. A motion-masked spatial pooling operator extracts the mean luminance ${\bar{L}}_{t}$ of the moving target:

(42) $${\bar{L}}_{t}=\frac{\sum_{(x,y)\in \Omega }F\left(x,y,t\right)\cdot I(|\Delta F\left(x,y,t\right)|\geq \tau_{m})}{\sum_{(x,y)\in \Omega }I(|\Delta F\left(x,y,t\right)|\geq \tau_{m})}$$

where ΔF is the frame difference. $\tau_{m}$ serves as a base threshold to filter minor illumination variations. I(·) represents the indicator function.

A sliding windowed difference calculates the temporal contrast variation $\Delta {\bar{L}}_{t}$. This operation compares the current observation window with the preceding reference window, both of length $N_{\mathrm{win}}$:

(43) $$\Delta {\bar{L}}_{t}=\frac{1}{N_{\mathrm{win}}}\sum_{\tau =0}^{N_{\mathrm{win}}-1}\left({\bar{L}}_{t-\tau }-{\bar{L}}_{t-N_{\mathrm{win}}-\tau }\right)$$

Accumulating $\Delta {\bar{L}}_{t}$ over the initial detection phase $T_{\mathrm{init}}$ determines the global channel polarity Γ∈{1,−1}:

(44) $$\Gamma =\mathrm{sgn}\left(\sum_{\tau \in T_{\mathrm{init}}}\Delta {\bar{L}}_{\tau }\cdot I(|\Delta {\bar{L}}_{\tau }\left|>\tau_{d}\right)\right)$$

where sgn(·) is the signum function, and $\tau_{d}$ acts as a calibrated decision threshold. A polarity of Γ=1 indicates a positive contrast trend, which activates the ON pathway and inhibits the OFF pathway. Conversely, Γ=−1 activates the OFF pathway. This unipolar configuration remains fixed throughout the subsequent inference phase.

#### 4.4.5. Adaptive Probability Mapping Strategy

Static probabilistic parameters restrict model adaptivity in dynamic environments. Consequently, an environment-aware empirical mapping function dynamically adjusts the synaptic transmission probability.

This function utilizes the environmental hyperparameters extracted from the initial frames: the spatial noise density $H_{\mathrm{space}}$ and the high-frequency jitter displacement $H_{\mathrm{jitter}}$. The formula defines the optimal transmission probability ${\mathrm{Prob}}_{\mathrm{opt}}$:

(45) $${\mathrm{Prob}}_{\mathrm{opt}}=\left\{\begin{array}{c} 1.0,\;\mathrm{if}\;H_{\mathrm{space}}\leq 0.15\;\mathrm{and}\;H_{\mathrm{jitter}}\leq \tau_{\mathrm{safe}}\\ {\mathrm{Prob}}_{\mathrm{base}}\cdot \exp\left(-\gamma_{\mathrm{space}}\cdot H_{\mathrm{space}}\right)+\gamma_{\mathrm{jitter}}\cdot \ln\left(1+H_{\mathrm{jitter}}\right),\;\mathrm{otherwise} \end{array}\right.$$

where the tolerance margin $\tau_{\mathrm{safe}}=0.5$ prevents non-structural pixel variations from erroneously triggering compensation. The parameters ${\mathrm{Prob}}_{\mathrm{base}}$, $\gamma_{\mathrm{space}}$, and $\gamma_{\mathrm{jitter}}$ represent the base probability, the spatial noise suppression coefficient, and the jitter compensation coefficient, respectively. Based on the quantitative calibration detailed in Section 5.2.2, these parameters are empirically set to ${\mathrm{Prob}}_{\mathrm{base}}=0.6139$, $\gamma_{\mathrm{space}}=0.791$, and $\gamma_{\mathrm{jitter}}=0.4334$.

Within this mechanism, the exponential term suppresses transmission to filter spatial noise. Simultaneously, the logarithmic term compensates for high-frequency jitter. Curve fitting based on quantitative ablation experiments calibrates the specific values of ${\mathrm{Prob}}_{\mathrm{base}}$, $\gamma_{\mathrm{space}}$, and $\gamma_{\mathrm{jitter}}$. To prevent total signal occlusion under extreme interference, a lower-bound threshold ${\mathrm{Prob}}_{\min}=0.4$ constrains the final output ${\mathrm{Prob}}_{\mathrm{final}}$:

(46) $${\mathrm{Prob}}_{\mathrm{final}}=\max\left({\mathrm{Prob}}_{\min},\min\left({\mathrm{Prob}}_{\mathrm{opt}},1.0\right)\right)$$

#### 4.4.6. Statistical Reliability Metrics

The incorporation of Gaussian random variables introduces random variance into the model outputs. To verify statistical stability and eliminate coincidental successes, the evaluation conducts N=50 independent repeated trials. Let $R_{n}$ denote the collision response peak ($\kappa_{\max}$) obtained in the *n*-th trial. The sample mean $\mu_{R}$ and standard deviation $\sigma_{R}$ quantify the output variations:

(47) $$\mu_{R}=\frac{1}{N}\sum_{n=1}^{N}R_{n}$$

(48) $$\sigma_{R}=\sqrt{\frac{1}{N-1}\sum_{n=1}^{N}{\left(R_{n}-\mu_{R}\right)}^{2}}$$

A smaller $\sigma_{R}$ reflects lower response variance under random disturbances.

The interquartile range (IQR) evaluates statistical stability while mitigating the influence of extreme outliers:

(49) $$\mathrm{IQR}=Q_{3}-Q_{1}$$

where $Q_{1}$ and $Q_{3}$ represent the 25th and 75th percentiles of the *N* peak values. A smaller IQR indicates a concentrated distribution of membrane potentials, reflecting the statistical stability of the probabilistic model.

To verify the reliability of the performance across experimental trials, the confidence interval (CI) for the peak membrane potentials is calculated. Assuming a normal distribution of the samples, the CI is formulated as:

(50) $$CI=\bar{x}\pm Z\frac{s}{\sqrt{n}}$$

where $\bar{x}$ represents the sample mean of the peak membrane potentials, *s* denotes the sample standard deviation, *n* is the total number of tested sequences, and *Z* represents the critical value from the standard normal distribution corresponding to the desired confidence level.

## 5. Results and Analysis

This section mainly evaluates the collision detection performance and interference suppression mechanism of the proposed model. Comparative experiments test the complete model incorporating adaptive probabilistic mechanisms and SRF, verify its robustness under mixed-interference environments, and confirm its performance advantages over existing baseline models. Structural ablation experiments further decompose the individual functions of feedback loops and probabilistic propagation in suppressing compound noise, so as to determine the appropriate position and regulation intensity of Gaussian random variables. At the end of this section, statistical analysis is performed to verify the consistency and stability of the model’s output signals after introducing random variables.

### 5.1. Comparative Experiments

In this section, the proposed model is compared with existing comparative models designed for specific interference: the spatial noise-robust models (Gaussian Prob-LGMD [23] and Bernoulli Prob-LGMD [12]) and an anti-jitter model featuring a dynamic temporal variance feedback loop regulated by B-spline sampling, hereafter referred to as F-B-LGMD [32]. The Prob-LGMD models mainly focus on filtering spatial noise. The F-B-LGMD suppresses high-frequency jitter under isolated, low-intensity spatial noise conditions, The model is challenged by simultaneous, high-intensity compound interference.

#### 5.1.1. Performance Evaluation Under Individual Noise Sources

To verify the proposed model’s ability to suppress incoherent background motion and detect looming threats under severe spatial noise (Gaussian and salt-and-pepper) or high-frequency jitter, it is compared with three models: the Gaussian Prob-LGMD, the Bernoulli Prob-LGMD, and the F-B-LGMD. To evaluate the robustness, two distinct scenarios are constructed using an approaching black micro-robot and an approaching white micro-robot. These setups investigate the model’s response stability to different interferences.

**Figure 2:**
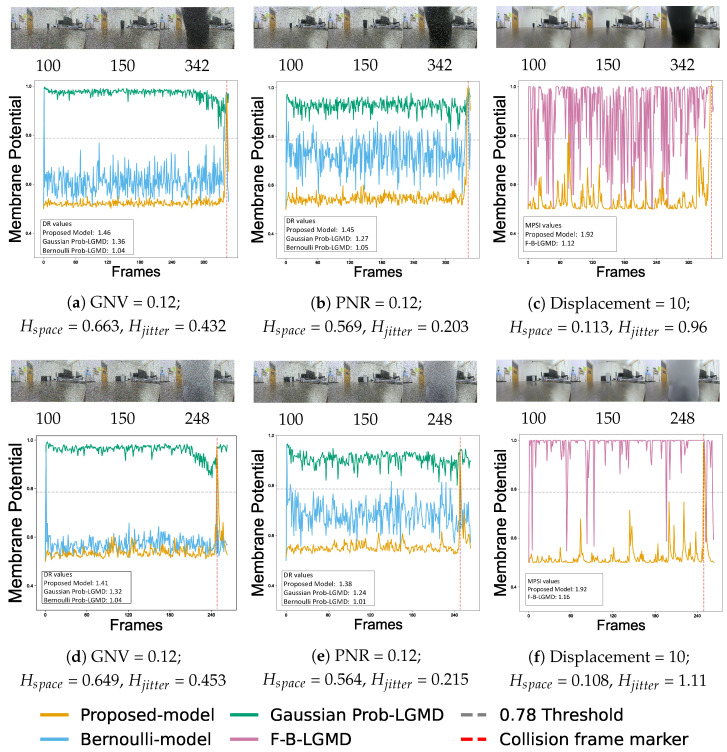
Membrane potential responses of looming black and white micro-robots under different isolated noise interference. (**a**,**d**) Gaussian noise interference (GNV = 0.12); (**b**,**e**) salt-and-pepper noise interference (PNR = 0.12); (**c**,**f**) displacement jitter interference (Displacement = 10). Among them, (**a**–**c**) correspond to the looming scenarios of the black micro-robot, and (**d**–**f**) correspond to those of the white micro-robot. All results are displayed at a approaching speed of 5 cm/s, showing the membrane potential response curves of different models under three challenging single-noise interferences. Here, $H_{space}$ and $H_{jitter}$ are the evaluation metrics for the model’s response under spatial noise and jitter interference, respectively.

Taking the black micro-robot scenario as an example, the test set comprised 36 video sequences across three approaching velocities (3, 5, and 7 cm/s) and six intensity levels of Gaussian and salt-and-pepper noise ranging from 0.07 to 0.12. Furthermore, to simulate the high-frequency jitter generated during the surface movement of the micro-robot, 21 additional sequences are generated by applying seven jitter levels ranging from 4 to 10 pixels to the same velocity configurations.As demonstrated in Figure 2, representative videos highlight the robustness of the models: a Gaussian noise variance GNV = 0.12, a salt-and-pepper noise ratio PNR = 0.12, and a 10-pixel jitter displacement.

In this evaluation, all comparative models adopt the optimal parameter settings for such scenarios as stated in their original literature. For the Gaussian Prob-LGMD model, the optimal parameters for daytime scenarios are used; the Prob value is set to 0.54 for the ON channel and 0.1 for the OFF channel. For the Bernoulli Prob-LGMD model, a Prob value of 0.5 is assigned. For the proposed model, a fixed Prob value of 0.52 is determined through experiments. This model shows reliable performance for collision detection in most scenarios.

As shown in Figure 2, the membrane potential responses of the models are compared under isolated noise and high-frequency jitter. Under isolated noise scenarios involving GNV = 0.12 or PNR = 0.12, the Bernoulli Prob-LGMD model is compromised; the overall baseline of its membrane potential is elevated, rendering it susceptible to false responses. Although the Gaussian Prob-LGMD model remains capable of identifying collisions, its response during non-collision periods is relatively high due to noise interference. Meanwhile, the membrane potential baseline of the proposed model remains consistently stable. The isolated noise does not induce discernible disturbances to the baseline, and the membrane potential exhibits an upward trend in response to the looming black micro-robot, demonstrating high noise resilience. Furthermore, under high-frequency jitter scenarios, the membrane potential of the F-B-LGMD model varies throughout the entire process with an elevated baseline, rendering it incapable of distinguishing between jitter noise and true collision stimuli. Meanwhile, the proposed model effectively suppresses background interference induced by jitter, maintaining a low baseline and generating valid responses solely to the looming target.

To objectively evaluate the robustness of the models, quantification is conducted using three metrics: SR, DR, and MPSI. Based on these metrics, the overall evaluation includes 114 video sequences. These videos feature black and white micro-robots looming at 3, 5, and 7 cm/s under different levels of Gaussian noise, salt-and-pepper noise, and high-frequency jitter. Across all 114 tests, the proposed model achieves an average SR of 85%. A portion of the comparative results is detailed in Table 3, Table 4 and Table 5. Furthermore, the model records an average DR above 1.3 and an average MPSI above 1.8. These quantitative findings corroborate that the proposed model reduces false responses during non-collision phases without compromising looming perception capabilities, proving to be more robust than the comparative models.

#### 5.1.2. Robustness Assessment Under Compound Interference

Building upon the isolated noise interferences, this study further evaluates the model’s robustness under composite spatial noise and compound interference (spatial noise coupled with high-frequency jitter) to simulate complex compound interference. Focusing on the looming black micro-robot scenario, a composite spatial noise test set is constructed by cross-combining the intensities of Gaussian noise (GNV ∈[0.01,0.12]) and salt-and-pepper noise (PNR ∈[0.01,0.12]). Three representative scenarios are established: (a) equal strong interference (GNV and PNR both ∈[0.06,0.12]); (b) Gaussian-dominated strong interference (GNV ∈[0.06,0.12], PNR ∈[0.01,0.05]); and (c) salt-and-pepper-dominated strong interference (the inverse configuration of scenario b). Each scenario within this compound interference test includes 75 test videos. All videos run at three approaching velocities (3, 5, and 7 cm/s, with 5 cm/s chosen for demonstration). This part has a total of 225 videos. Parameter settings for the comparative models remain the same as mentioned above.

**Figure 3:**
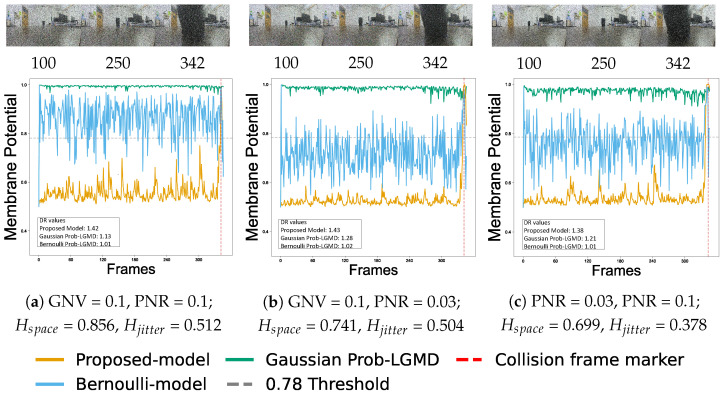
Collision detection results of different models under compound interference of spatial noise. (**a**) Equal-intensity composite spatial noise (GNV = 0.1, PNR = 0.1); (**b**) Gaussian-dominated composite spatial noise (GNV = 0.1, PNR = 0.03); (**c**) Salt-and-pepper-dominated composite spatial noise (GNV = 0.03, PNR = 0.1). The figure compares the membrane potential response characteristics of different models under compound spatial interference.

The membrane potential responses in Figure 3a–c indicate that both the Gaussian and Bernoulli Prob-LGMD models exhibit pronounced baseline variations during the non-looming phase, increasing the risk of false responses. By comparison, the proposed model maintains a globally stable baseline across all three interference scenarios, effectively capturing looming characteristics despite the compound interference. To objectively evaluate the model under these compound interference, the test suite utilizes 231 video sequences depicting a black micro-robot looming at velocities of 3, 5, and 7 cm/s. Quantitative analysis corroborates the overall stability of the model across all scenarios, yielding a SR of 88%, a mean DR of 1.38. A detailed subset of the comparative experimental results is provided in Table 3 and Table 4.

**Figure 4:**
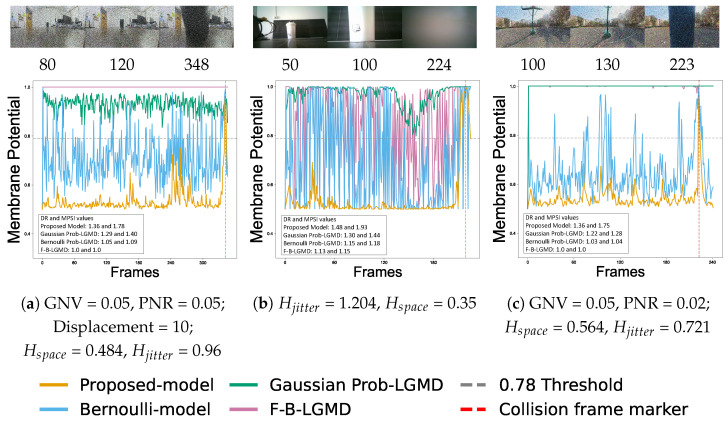
Collision detection results of different models under compound interference of spatial noise and high-frequency jitter. (**a**) Synthetic compound interference scenario with 10-pixel high-frequency jitter and equal compound noise of GNV = 0.05 and PNR = 0.05; (**b**) Videos captured by the micro-robot at three different velocities, displayed at the stable speed of 7 cm/s with 8–10 pixel high-frequency jitter; (**c**) These real jitter videos are captured by a handheld camera. The scenario features complex backgrounds and natural jitter, with weak compound noise added at GNV = 0.05 and PNR = 0.02. The figure compares the membrane potential response characteristics of different models under multi-source coupled disturbance.

The evaluation is further extended to compound interference, covering three gradual scenarios as shown in Figure 4a–c: (a) synthetic compound interference with GNV ∈[0.01,0.05], PNR ∈ [0.01,0.05] and jitter ranging from 8 to 10 pixels. Given the intense high-frequency jitter in this group, the amplitude of spatial noise is set to a relatively low level. This group includes 225 videos; (b) videos of an approaching white water cup, captured by the micro-robot at three velocities. This group includes 30 videos. The group at 7 cm/s is selected for display, as this speed generates high-frequency jitter of 8 to 10 pixels and brings prominent interference; and (c) these real jitter videos are captured by the camera. Considering that the camera possesses inherent noise filtering capabilities, additional spatial noise is injected to construct compound interference. Due to the background clutter already present in the scene, the level of the injected spatial noise is set relatively low, with GNV ∈[0.01,0.05] and PNR ∈[0.01,0.05]. This test group contains a total of 25 videos.

As observed in Figure 4a–c, the Bernoulli Prob-LGMD, Gaussian Prob-LGMD, and F-B-LGMD models experience severe baseline elevation and oscillations under these compound interference, generating numerous false responses. Meanwhile, the proposed model limits baseline shifts and displays a steady upward trajectory during the target approach, successfully crossing the warning threshold. Performance metrics across the compound interference further validate this: The constructed evaluation suite comprises a total of 280 video sequences tested under these varying scenarios. Across all tests, quantitative analysis corroborates the overall stability of the model, yielding a mean SR of 81%, a mean DR of 1.39, and a mean MPSI of 1.81. A portion of the comparative experimental results is detailed in Table 3, Table 4 and Table 5. While combined noise and jitter exacerbate background stimuli and reduce detection rates compared to isolated noise, the proposed model remains robust under compound interference, ensuring reliable collision threat identification.

### 5.2. Ablation Studies and Mechanism Analysis

To clarify the contribution of each structural module in reducing compound interference, this section presents ablation studies evaluating the independent and compound interference resilience of the Spatial Residual Feedback and the Gaussian Random Variable. Guided by these evaluations, the effective placement of random variables within the network and the appropriate configuration of probabilistic parameters are determined.

#### 5.2.1. Ablation Study of the Mechanisms

To verify the independent functions and synergistic effects of the Spatial Residual Feedback (SRF) jitter suppression module and the Gaussian Random Variable (GRV) noise filtering module, a mechanism ablation study is conducted. The evaluation adopts two groups of real-world interference video sequences: (a) Pedestrian looming captured by a fixed camera under compound spatial noise. GNV ∈ [0.01,0.05] and PNR ∈ [0.01,0.05], with the adopted values set to 0.05 for both parameters. This part includes 25 video sequences; (b) High-frequency jitter footage captured by a handheld camera; (c) Natural jitter from handheld shooting superimposed with compound spatial noise, where GNV = 0.05 and PNR = 0.02. Groups (b) and (c) contain a total of 26 video sequences.

The membrane potential responses are compared across four model variants for ablation analysis: the intact model, the variant lacking the SRF module (without SRF), the variant lacking the GRV module (without GRV), and the variant lacking both modules (without SRF & GRV).

**Figure 5:**
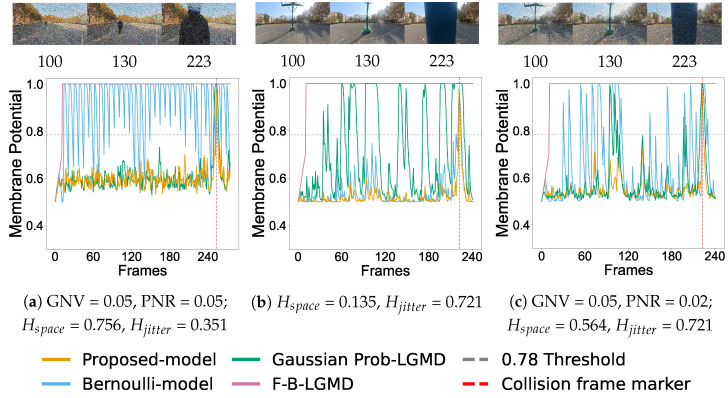
Membrane potential responses of the proposed model and its ablation variants under combined spatial noise interference in the approaching scenario. (**a**) Pedestrian looming captured by a fixed camera under compound spatial noise of GNV = 0.05 and PNR = 0.05; (**b**) High-frequency jitter video captured by a handheld camera; (**c**) High-frequency jitter video captured by a handheld camera, superimposed with compound spatial noise added at GNV = 0.05 and PNR = 0.02.

The membrane potential curves in Figure 5a–c illustrate the respective robustness disparities among the variants. The variant lacking both modules exhibits deficiencies under compound spatial interference. Across scenarios (a)–(c), its membrane potential prematurely saturates to 1.0 during the initial testing phases. Meanwhile, it yields no effective response, failing to distinguish background interference from true looming signals. Although the without SRF model filters out a portion of the spatial noise via the GRV module, it is unable to suppress high-frequency jitter. As a result, in the jitter-inclusive scenarios (b) and (c), its response curves display large variations, generating numerous false peaks that exceed the 0.78 warning threshold. The without GRV model loses its capacity for spatial noise suppression, causing instant membrane potential saturation in scenarios (a) and (c), even though its SRF module still manages to suppress jitter in scenario (b).

The synergy between Gaussian random variables and spatial residual feedback provides a reliable scheme for micro-robots navigating compound interferences. While the current study confirms the efficacy of unipolar filtering through a priori contrast polarity initialization, subsequent work will investigate the automatic switching between ON and OFF channels during real-time operation. In addition, future studies will evaluate the deployment feasibility of the model in micro-robot applications to facilitate online hardware validation.

The findings demonstrate that a single module is inadequate for mitigating concurrent jitter and spatial noise. The jitter suppression provided by the SRF functionally complements the discrete noise filtering of the GRV module. The absence of either module results in a substantial degradation in the overall robustness of the model. Effective collaboration between the two structures remedies the drawbacks of individual modules, allowing the model to produce reliable collision warning signals under compound interference.

#### 5.2.2. Parameter Optimization of the Random Variable

To enhance the robustness of the model in compound interference, this section systematically calibrates the probabilistic parameters and constructs an adaptive strategy. Because the ON and OFF pathways selectively respond to light and dark looming targets, a single polarity channel dominates perception in a fixed scenario. Relying exclusively on this dominant channel prevents the introduction of irrelevant background noise from the non-dominant pathway. Consequently, based on the prior luminance information defined in Section 4.4.4, the non-dominant pathway is deactivated. This operation circumvents redundant interference and ensures that probabilistic optimization focuses on the effective signal pathway, thereby facilitating deployment on resource-constrained micro-robot platforms.

**Figure 6:**
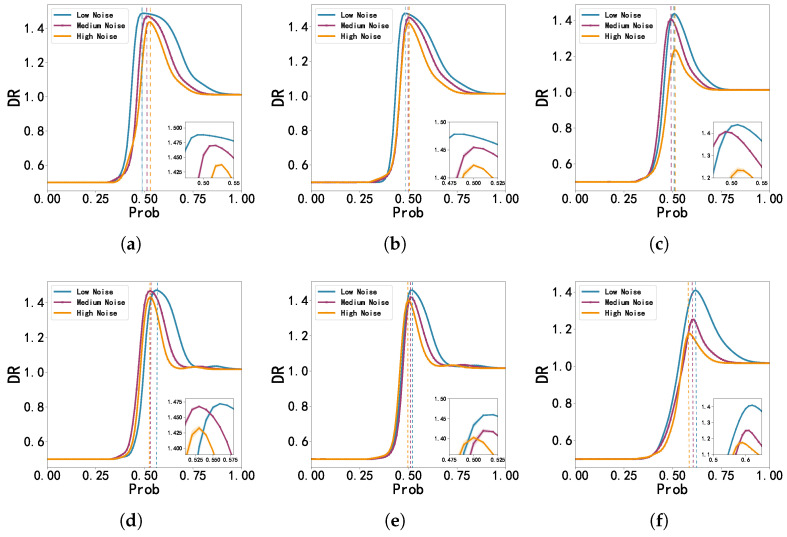
Optimization curves of transmission probability (Prob) under spatial noise interference. The curves illustrate how DR varies with Prob at different noise intensities for black and white micro-robots. (**a**) DR under isolated Gaussian noise for the black micro-robot; (**b**) DR under isolated salt-and-pepper noise for the black micro-robot; (**c**) DR under combined spatial noise for the black micro-robot; (**d**) DR under isolated Gaussian noise for the white micro-robot; (**e**) DR under isolated salt-and-pepper noise for the white micro-robot; (**f**) DR under combined spatial noise for the white micro-robot. The dashed vertical lines indicate the optimal probability values corresponding to the peak DR for each noise intensity tier.

As shown in Figure 6, the influence of probability parameters on model performance is systematically evaluated. The experimental dataset comprises videos of micro-robots looming at three distinct velocities (3, 5, and 7 cm/s). Figure 6a,b,d,e depict performance under isolated spatial noise sources, such as Gaussian and salt-and-pepper noise. For each noise intensity tier, namely Low ∈ [0.01,0.04], Medium ∈ [0.05,0.08], and High ∈ [0.09,0.12], we include four specific levels. Thus, each curve is derived from 12 test sequences (4 noise levels × 3 velocities). Figure 6c,f depict performance under combined spatial noise, where Gaussian and salt-and-pepper noise are superimposed with equal intensity, such as 0.01 plus 0.01 for the Low tier. These composite conditions also follow the same 4-level noise distribution across three velocities, resulting in 12 test sequences per curve.

**Figure 7:**
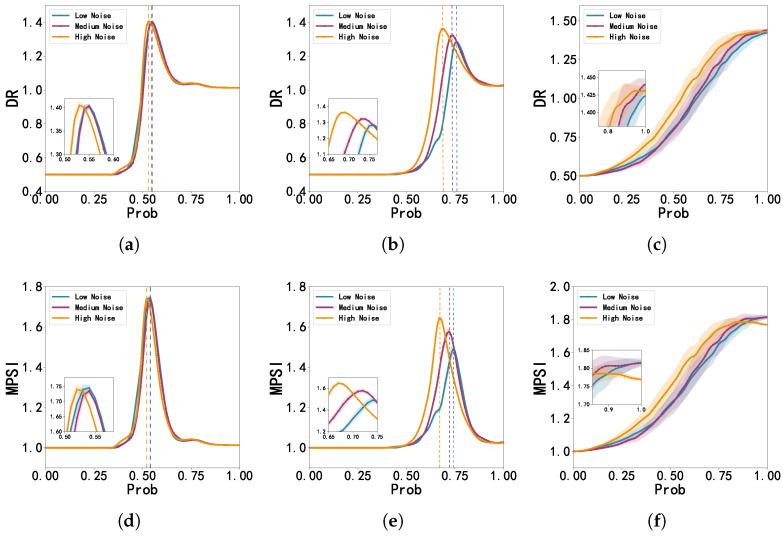
Optimization curves of transmission probability (Prob) under compound interferences (spatial noise and high-frequency jitter). The curves illustrating how DR and MPSI vary with Prob for black and white micro-robots. (**a**) DR results for the black micro-robot with fixed spatial noise (GNV = 0.075, PNR = 0.075); (**b**) DR results for the white micro-robot with fixed spatial noise (GNV = 0.05, PNR = 0.05); (**c**) DR results for the black micro-robot following multi-layer probabilistic regulation; (**d**) MPSI results for the black micro-robot with fixed spatial noise (GNV = 0.075, PNR = 0.075); (**e**) MPSI results for the white micro-robot with fixed spatial noise (GNV = 0.05, PNR = 0.05); (**f**) MPSI results for the black micro-robot following multi-layer probabilistic regulation. The dashed vertical lines indicate the optimal probability values corresponding to the peak performance metrics for each jitter amplitude tier.

As illustrated in Figure 7, the test scenarios involve compound interference, where spatial noise is superimposed on high-frequency jitter. For these experiments, the spatial noise intensities are fixed at GNV = 0.075 and PNR = 0.075 for the black micro-robot, and at GNV = 0.05 and PNR = 0.05 for the white micro-robot. The jitter amplitude is modulated across three tiers: [1,3], [4,6], and [7,10] pixels. To ensure statistical representativeness, the Low and Medium jitter tiers consist of 9 test sequences (3 levels × 3 velocities), while the High tier consists of 12 test sequences (4 levels × 3 velocities), with each curve representing the averaged performance metrics derived from these varying sequence counts. Figure 7a,d present the DR and MPSI results for the black micro-robot, while Figure 7b,e illustrate the corresponding results for the white micro-robot. Figure 7c,f depict the performance of the black micro-robot following multi-layer probabilistic regulation. In these experiments, after selecting the appropriate polarity channel (ON or OFF) and optimal parameters within the channel, we activate the stochastic variables in the synaptic transmission pathways between the medulla and grouping layers (S to G), and between the grouping and LGMD layers (G to LGMD), to further evaluate the impact of multi-layer probabilistic regulation on DR and MPSI.

Under the single-pathway activation premise, the transmission probability is evaluated within the [0,1] interval at a resolution of 0.01. The resulting DR and MPSI metrics exhibit an inverted U-shaped distribution, indicating a distinct effective interval. Across all evaluated interference scenarios, the effective probability clusters within [0.45,0.60] for the black micro-robot and within [0.50,0.65] for the white micro-robot. Based on these empirical observations, we calibrated the coefficients in Equation (42) by extracting the optimal probability values corresponding to the peak MPSI under varying noise intensities. Using non-linear least squares optimization to minimize the empirical error, the parameters for the active channel are determined as follows: the base probability ${\mathrm{Prob}}_{\mathrm{base}}=0.6139$, the spatial suppression coefficient $\gamma_{\mathrm{space}}=0.791$, and the jitter compensation coefficient $\gamma_{\mathrm{jitter}}=0.4334$.

Notably, as observed in Figure 7c,f, once the effective medulla layer probability is established, modulating the synaptic random variables in subsequent layers (S-to-G and G-to-LGMD) fails to yield further noise-reduction gains. For example, under compound interference (GNV = 0.075, PNR = 0.075, 10-pixel jitter) for the black micro-robot, enabling multi-layer probabilistic regulation leads to a decline in membrane potential amplitude. The metrics can only return to their original levels when the probability is set to 1. This confirms that redundant probabilistic intervention at posterior stages excessively weakens the continuous physical contours of real targets, precipitating a degradation in overall response intensity rather than enhancing robustness.

**Figure 8:**
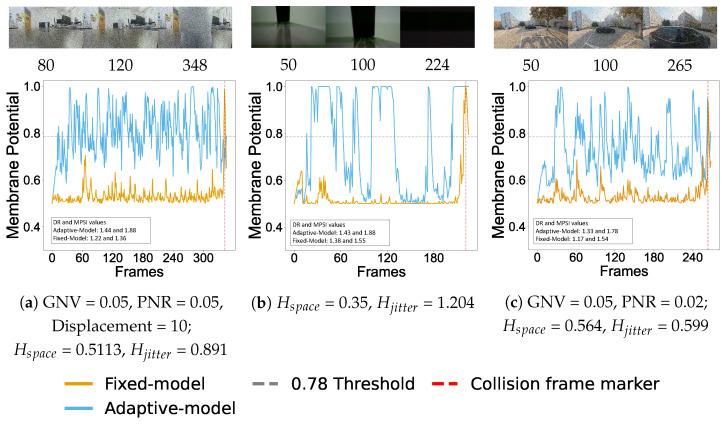
Comparative membrane potential responses of the fixed and adaptive models subjected to compound interference. (**a**) Simulated sequence featuring a white micro-robot looming under 10-pixel mechanical jitter and equal spatial noise (GNV = 0.05, PNR = 0.05); (**b**) Video sequence captured by the micro-robot operating at a velocity of 7 cm/s toward a black water cup; (**c**) Real jitter video sequence captured by a handheld camera depicting an approach toward a black car, with weak compound noise added at GNV = 0.05 and PNR = 0.02.

To validate the practical performance of the adaptive probability strategy, comparative evaluations are conducted against the fixed probability parameters model (Prob=0.52) to demonstrate its robustness in unknown compound interference scenarios. The constructed test suite comprises 280 independent video sequences entirely excluded from prior parameter calibration. Taking the synthetic interference scenario as an example, the subset includes 225 sequences featuring a white micro-robot, with Gaussian and salt-and-pepper noise intensities ranging from 0.01 to 0.05 and jitter amplitudes ranging from 8 to 10 pixels. Furthermore, to evaluate real-world generalization, 30 sequences of a micro-robot approaching a black cup across three velocities (3, 5, and 7 cm/s) and 25 handheld sequences of a looming black car superimposed with synthetic noise are incorporated. As demonstrated in Figure 8a–c, representative videos highlight these scenarios: a 5 cm/s approach with GNV = 0.05, PNR = 0.05, and a 10-pixel jitter; a 7 cm/s real-world approach; and a handheld approach with GNV = 0.05 and PNR = 0.02, respectively.

**Table 3:** SR for different models.

Scenario	Bernoulli-Prob-LGMD	Gaussian-Prob-LGMD	F-B-LGMD	Proposed
Figure 2a	67	78	72	83
Figure 2b	67	72	67	83
Figure 2c	62	71	76	86
Figure 2d	72	78	72	89
Figure 2e	67	78	72	83
Figure 2f	62	71	76	86
Figure 3a	64	72	68	87
Figure 3b	68	76	71	89
Figure 3c	65	73	68	88
Figure 4a	61	68	74	82
Figure 4b	57	67	73	80
Figure 4c	56	64	68	80
Scenario	fixed-model	adaptive-model	—	—
Figure 8a	60	80		
Figure 8b	53	77		
Figure 8c	50	73		

**Table 4:** DR for different models.

Scenario	Bernoulli-Prob-LGMD	Gaussian-Prob-LGMD	F-B-LGMD	Proposed
Figure 2a	1.04	1.30	–	1.40
Figure 2b	1.05	1.27	–	1.45
Figure 2d	1.04	1.32	–	1.41
Figure 2e	1.01	1.24	–	1.38
Figure 3a	1.01	1.13	–	1.42
Figure 3b	1.02	1.28	–	1.43
Figure 3c	1.01	1.21	–	1.36
Figure 4a	1.05	1.29	1.00	1.36
Figure 4b	1.15	1.30	1.13	1.48
Figure 4c	1.03	1.22	1.00	1.36
Scenario	fixed-model	adaptive-model	—	—
Figure 8a	1.22	1.44		
Figure 8b	1.38	1.43		
Figure 8c	1.17	1.33		

**Table 5:** MPSI for different models.

Scenario	Bernoulli-Prob-LGMD	Gaussian-Prob-LGMD	F-B-LGMD	Proposed
Figure 2c	–	–	1.12	1.92
Figure 2f	–	–	1.16	1.92
Figure 3a	1.02	1.14	1.07	1.65
Figure 3b	1.05	1.11	1.10	1.71
Figure 3c	1.01	1.09	1.08	1.64
Figure 4a	1.09	1.40	1.00	1.78
Figure 4b	1.18	1.44	1.16	1.93
Figure 4c	1.04	1.28	1.00	1.75
Scenario	fixed-model	adaptive-model	—	—
Figure 8a	1.36	1.81		
Figure 8b	1.55	1.85		
Figure 8c	1.54	1.78		

In these evaluations, the adaptive model computes target probabilities ($Prob_{opt}$) of 0.635, 0.512, and 0.579 for the three representative scenarios. During the non-collision phase, the fixed model exhibits severe membrane potential variations due to compound interference, frequently approaching the 0.78 warning threshold and generating false responses. Conversely, the adaptive model utilizes prior environmental estimations to constrain the transmission probability, effectively stabilizing the membrane potential at a lower range during safe intervals. Upon reaching the collision frame, both configurations successfully execute a sharp surge in membrane potential to trigger the warning response.

Quantitative metrics corroborate this enhanced performance (Table 3, Table 4 and Table 5). Across the 280 test sequences, the adaptive model yields an average SR of 82%, a mean DR of 1.40, and a mean MPSI of 1.81. These results confirm that the adaptive modulation strategy effectively mitigates novel compound interferences, demonstrating stronger background suppression and temporal stability compared to the fixed probability parameters architecture.

### 5.3. Statistical Stability Analysis

Given the inherent stochastic nature of the proposed synaptic transmission mechanism, rigorous statistical validation is imperative to verify the stability of the model. To achieve this, we evaluate the output consistency across four distinct operational scenarios. Scenario (a) establishes a noise-free baseline, specifically evaluated using a representative looming sequence of the black micro-robot approaching at a constant velocity of 5 cm/s. Scenarios (b) through (d) introduce varying degrees of unknown compound interferences, corresponding to the complex testing environments depicted in Figure 8.

To ensure statistical significance and adequately account for internal algorithmic randomness, each scenario is subjected to 50 independent repeated trials. This sample size (N=50) provides a robust statistical foundation for analyzing the probabilistic distribution of the outputs. Across all repeated trials, the membrane potentials generated by the proposed model maintain strict consistency and stability, demonstrating reliable alerting capabilities without succumbing to random stochastic variations.

**Figure 9:**
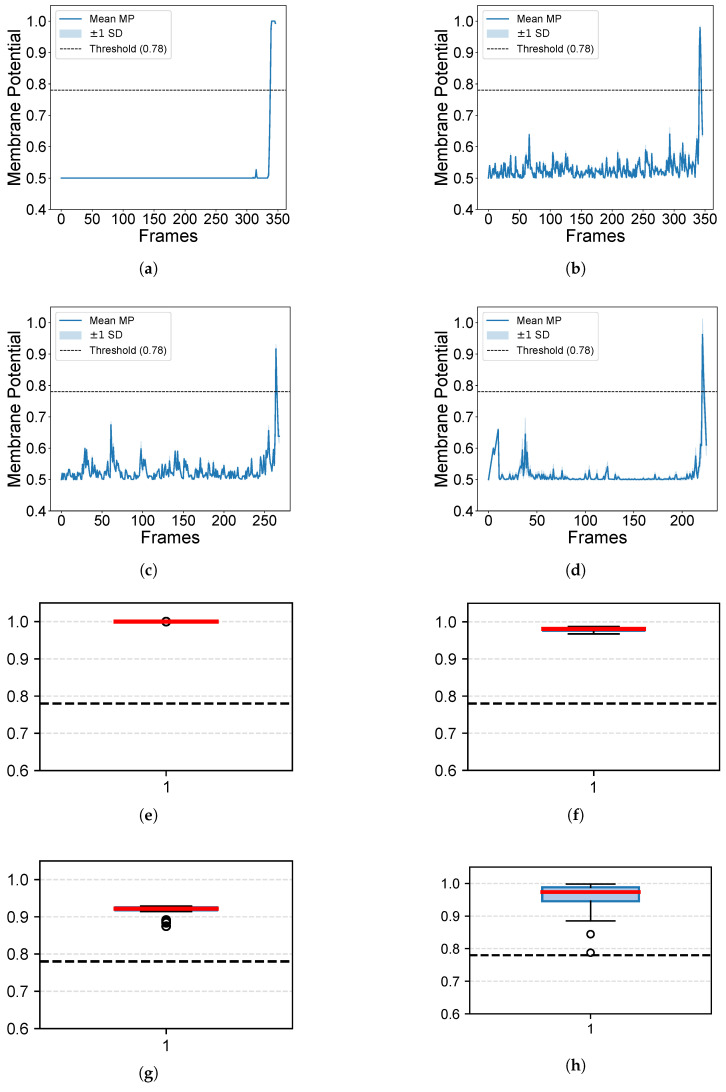
Statistical results of the membrane potential to verify the stability of the proposed model under four scenarios, with 50 repeated tests conducted for each scenario. The top row (**a**–**d**) shows the confidence envelopes (mean ± standard deviation, SD) of the membrane potential under each scenario, while the bottom row (**e**–**h**) shows the corresponding boxplots. The four scenarios are defined as follows: (**a**) Noise-free scenario: black micro-robots approaching at 5 cm/s (**b**–**d**) Compound interference scenarios corresponding to the three different cases presented in Figure 8. In the boxplots (**e**–**h**), the solid red line indicates the median value, the blue box represents the interquartile range (IQR), the whiskers denote the data range within 1.5 times the IQR, and the open circles represent outlier data points. The black dashed line across all subfigures represents the predefined warning threshold of 0.78.

As illustrated by the confidence envelopes in Figure 9a–d, the SRF circuit and GRV module exert synergistic suppression during non-collision phases. The membrane potentials corresponding to all test videos stay steadily below the predefined warning threshold of 0.78, with no false responses occurring throughout the tests. Even when exposed to superimposed high-density spatial noise and high-frequency jitter, the model stably extracts the looming signatures of targets. As shown in the box plots of Figure 9e–h, the peak membrane potentials are concentrated above 0.85 under severe compound interferences.

To rigorously validate these observations, Table 6 details the descriptive statistics across the four evaluated scenarios. The quantitative metrics reveal that the standard deviations ($\sigma_{R}$) and interquartile ranges (IQRs) remain robustly constrained, even under intense mechanical jitter (Scenario c). Moreover, the 95% confidence intervals (CIs) confirm that the peak membrane potentials strictly reside above the 0.78 threshold across all interference conditions. A one-sample Wilcoxon signed-rank test further verifies that the median peak responses significantly exceed this predefined threshold (p<0.001). These statistical validations demonstrate that the proposed stochastic framework maintains consistent and reliable early warning capabilities under complex compound interferences, without succumbing to coincidental variations.

## 6. Further Discussion

The experimental results demonstrate that the effect of compound interference on visual collision detection is more severe than the simple superposition of individual noises. This phenomenon stems from the interaction between global spatial shifts and random discrete pixels. While high-frequency jitter shifts background edges to cause continuous changes in local brightness, discrete noise introduces random, isolated pixel variations to the image. When combined, the randomly distributed noise pixels break the continuous moving edges caused by the jitter. This divides the smooth brightness transitions into small, irregular fragments. These sharp brightness changes resemble the visual expansion of an approaching target, causing the network to generate false responses.

Existing comparative models generally adopt fixed probability parameters and lack adaptive mechanisms, making them unable to cope with disturbances over a wide range. Because they have no feedback regulation, the feedforward variants among these models lead to continuous error accumulation across layers. Under such compound interference with jitter, the normal operation of these models is affected. This triggers rapid saturation of membrane potential responses and renders the models not workable.

The proposed model maintains stable SR, DR, and MPSI metrics under compound interference. This reliability is attributed to the sequence-level cooperation between the SRF and the probabilistic mechanisms. During the initial processing phase between the photoreceptor layer and the parallel ON/OFF channels, the SRF extracts feature residuals. By suppressing the variations caused by jitter, this step successfully isolates the compound interference into discrete noise. Relying on prior target luminance information, the model subsequently executes a contrast polarity initialization scheme to statically activate the matching ON or OFF pathway, reducing compound interference. For this retained active channel, the model calculates effective probability parameters based on evaluated environmental hyperparameters, enabling robust noise filtering.

This study explored the appropriate placement of random variables within the visual network. Under the evaluated compound interference conditions, ablation experiments indicate that placing normally-distributed random variables within the front-end bipolar pathways is sufficient to achieve substantial noise reduction. Subsequent comparative trials verify that introducing random variables into later computational stages (e.g., from the S layer to the G layer or the G layer to the LGMD layer) provides no additional noise-reduction gains. Applying Gaussian random variables across multiple layers disrupts the continuous spatial contours of the target and attenuates the overall network response.

Consequently, the random variable mechanism in subsequent computational layers acts merely as a structural redundancy within the current experimental contexts. Under the evaluated conditions, the SRF and the probabilistic regulation of the unipolar channel effectively offset the majority of the noise. However, if the visual input introduces uncharacterized disturbances, such as localized dynamic light spots or abrupt non-uniform illumination changes, the current mechanisms may be insufficient. These photometric disturbances are fundamentally different from the stochastic signal variations and coherent motion shifts addressed in this study. While our model focuses on mitigating stochastic spatial noise and high-frequency jitter, which are inherent signal-level fluctuations, the disturbances mentioned above act as extrinsic photometric modulations that fundamentally alter the underlying contrast distribution of the visual input. Because these represent a structural change in the intensity mapping rather than simple signal noise, they fall outside the scope of our current signal processing framework. In such novel scenarios, whether activating random variables in subsequent computational stages (such as the G layer) could provide additional robustness for feature extraction remains to be verified. Exploring how these random variables can be adapted to mitigate the aforementioned photometric disturbances will serve as a crucial direction for our future research, allowing us to extend the model’s capabilities beyond stochastic signal noise to more complex visual environments.

## 7. Conclusions

This paper proposes an LGMD-inspired collision detection model adopting normally distributed Gaussian random variables and spatial residual feedback (SRF) to address the compound interference of spatial noise and high-frequency jitter. By placing random variables between the excitation and local summation layers to filter spatial noise and employing the SRF to suppress high-frequency jitter, the robustness of the model is enhanced. Numerous repeated experiments across synthetic and real-world video sequences demonstrate that the proposed architecture reduces false responses during non-collision phases, exhibiting higher stability than existing comparative models.

Ablation studies confirm that these two mechanisms functionally complement each other to achieve optimal noise reduction. Experimental findings indicate that applying a contrast polarity initialization scheme during the pre-processing stage allows the model to selectively activate the ON or OFF pathway based on the target’s luminance characteristics. This approach effectively filters spatial noise by tuning the synaptic transmission probability of the retained unipolar channel and reducing computational overhead. Once the parameters for this active channel are established, introducing redundant random variables in subsequent computational layers yields no further noise-reduction gains; instead, it dilutes valid target spatial contours and weakens the overall response intensity.

To translate these findings into robust applicability, an empirical parameter mapping function is constructed. This formulation enables the model to adjust the transmission probability based on evaluated environmental hyperparameters for spatial noise and jitter ($H_{space}$ and $H_{jitter}$), offering an alternative to fixed probability parameters. Data from repeated statistical experiments confirm that this adaptive probability-tuning mechanism restricts output instabilities and maintains stable DR and MPSI metrics under complex compound interference, ensuring consistent and reliable alert signals.

The synergy between adaptive Gaussian random variables and spatial residual feedback provides a reliable scheme for micro-robots navigating compound interference.

By effectively decoupling and filtering high-frequency jitter and spatial noise, the proposed model ensures robust looming perception, substantially enhancing the practical viability of LGMD-inspired visual systems in complex environments. Moving forward, our future work will primarily focus on deploying this complete visual pipeline onto physical micro-robots for closed-loop experiments, alongside further algorithmic optimizations for diverse motion patterns and photometric disturbances.

## Figures and Tables

**Table 1:** Model Parameters.

Parameter	Description	Value
$\alpha_{1,2}$	The control coefficient of inhibitory flow	0.1 and 0.85
$\sigma_{1,2}$	The Gaussian spatial convolution kernel	0∼1
$T_{s}$	Threshold in G layer processing	875
$C_{\omega }$	Coefficient in G layer processing	70∼100
prob	Probability parameter	adaptable
δ	The adaptation rate for the dynamic baseline	0.2
β	The momentum decay coefficient	0.85
*g*	Amplification gain parameter	50∼70
{C,R}	Spatial dimension of input stimuli	320 × 240

**Table 2:** Configuration of the Visual Testing Dataset.

Subset	Environment	Experimental Conditions	Totals
I	Subset 1	Synthetic Noise (Gaussian & salt-and-pepper: 0.01–0.12; jitter: 1–10 px)	4200
II	Subset 2	mechanical jitter (2–3 px, 4–6 px, 8–10 px)	60
III	Subset 3	dynamic background + mixed Noise (0.01–0.06)	76

**Table 6:** Descriptive Statistics and Reliability Metrics Across 50 Independent Trials.

Test Scenario	Mean ($\mu_{R}$)	Std. Dev. ($\sigma_{R}$)	IQR	95% CI	*p*-Value
(a) Ideal Baseline Scenario	0.999	0.002	0.000	[0.998,1.000]	<0.001
(b) Synthetic Compound Interference	0.978	0.007	0.010	[0.976,0.980]	<0.001
(c) Real Micro-robot Locomotion	0.959	0.048	0.049	[0.946,0.973]	<0.001
(d) Real Handheld Outdoor Jitter	0.917	0.013	0.006	[0.913,0.920]	<0.001

## Data Availability

The original contributions presented in this study are included in the article. Further inquiries can be directed to the corresponding author.
